# Preliminary assessment of genetic variation in the Japanese endemic freshwater crab, *Geothelphusadehaani*, based on mitochondrial DNA sequences

**DOI:** 10.3897/BDJ.11.e97438

**Published:** 2023-06-27

**Authors:** Joana Joy De la Cruz Huervana, Yuichi Kano, Daiki Ando, Norio Onikura, Yoshihisa Kurita

**Affiliations:** 1 Fishery Research Laboratory, Kyushu University, Fukuoka, Japan Fishery Research Laboratory, Kyushu University Fukuoka Japan; 2 Aquaculture Department, Southeast Asian Fisheries Development Center, Iloilo, Philippines Aquaculture Department, Southeast Asian Fisheries Development Center Iloilo Philippines; 3 Kyushu University, Fukuoka, Japan Kyushu University Fukuoka Japan; 4 Kyushu Open University, Fukuoka, Japan Kyushu Open University Fukuoka Japan

**Keywords:** mitochondrial DNA, COI, cytB, DNA sequences, *Geothelphusa*, freshwater crabs, endemic, phylogenetic analysis, genetic diversity

## Abstract

*Geothelphusadehaani*, a freshwater crab species endemic to Japan, has the largest distribution range amongst the 19 known species in the country. Due to its low dispersal capability and restricted habitat to freshwater, it serves as an excellent model for understanding gene flow between geographically isolated populations. In this study, we analysed the genetic relationships of 26 *G.dehaani* populations collected from different locations in the Japanese archipelago using two mitochondrial DNA regions - cytochrome oxidase subunit I (*COI)* and cytochrome b (*cytB*). Our results from the analysis of molecular variance (AMOVA) revealed high genetic variation amongst populations and the phylogenetic analysis identified four geographical groups: Clade I - Honshu and Shikoku, Clade II - north-eastern Kyushu, Clade III - southern Kyushu and Clade IV - north-western Kyushu. Notably, Clade IV exhibited the highest genetic distance amongst the observed groupings. These findings highlight the need for further examination of *G.dehaani* in Kyushu, including morphological and behavioural traits, to better understand the observed diversity within the species in the region.

## Introduction

Freshwater crabs can be found in a wide range of aquatic environments, including rivers, streams and lakes. Certain species require specific habitat conditions that may limit their range, whilst others are more tolerant of various habitat types, which may result in their widespread geographic distribution (Cumberlidge and Ng 2010). Freshwater crabs of the genus *Geothelphusa* are found in the East Asian Island Arc, which stretches from Taiwan, Ryukyus and the main islands of Japan (Shih and Ng 2011; Shy et al. 2020). Nineteen species of *Geothelphusa* have been identified in Japan, including *G.dehaani* (White 1847), *G.obtusipes* (Stimpson 1858), *G.levicervix* (Rathbun 1898), *G.sakamotoana* (Rathbun 1905), *G.tenuimanus* (Miyake and Minei 1965), *G.aramotoi* (Minei 1973), *G.exigua* (Suzuki and Tsuda 1994), *G.minei* (Shy and Ng 1998), *G.shokitai* (Shy and Ng 1998), *G.marmorata* (Suzuki and Okano 2000), *G.miyakoensis* (Shokita et al. 2002), *G.marginatafulva* (Naruse et al. 2004), *G.marginatamaruginata* (Naruse et al. 2004), *G.grandiovata* (Naruse et al. 2006), *G.iheya* (Naruse et al. 2006), *G.kumejima* (Naruse et al. 2006), *G.amagui* (Naruse and Shokita 2009), *G.koshikiensis* (Suzuki and Kawai 2011) and *G.mishima* (Suzuki and Kawai 2011). From these, only five species are known north of the Ryukyus: *G.exigua* in Osumi Peninsula, *G.marmorata* in Yakushima Island, *G.koshikiensis* in Koshiki Islands, *G.mishima* in Mishima Islands and *G.dehaani* from northernmost Ryukyus to Hokkaido (Suzuki and Tsuda 1994; Suzuki and Okano 2000; Suzuki and Kawai 2011; Sugime et al. 2022). *Geothelphusadehaani* has the most widespread distribution and is the only known species that can be found in localities with colder climates as other species are concentrated in warmer freshwater habitats (Shih and Ng 2011). These crabs only produce a few large eggs and once fertilised, the eggs develop directly into juvenile crabs, without a planktonic larval stage (Yamaguchi and Takamatsu 1980; Hartnoll 1988). Likewise, the migratory abilities of freshwater crabs are relatively weak since they are intolerant of brackish water and marine environments (Shy et al. 2020). With such characteristics, *G.dehaani* has low dispersal capability and geographical isolation amongst its populations can be expected. These factors also indicate low levels of genetic variation within populations and high levels of genetic variation between populations. As a result, *G.dehaani* can be a good model to study levels of gene flow between geographically isolated populations with a wide distribution range. Similarly, freshwater decapods are good indicators to reflect the connection between genetic isolation and past geological events (Okano et al. 2000b; Suzuki 2001; Shih et al. 2004; Shih et al. 2006; Shih et al. 2011).

Previous studies to clarify the genetic relationships amongst *G.dehaani* populations used allozyme markers as a genetic tool and were only conducted in short geographic ranges; Okano et al. (2000b) worked in Kagoshima mainland in Kyushu and its neighbouring islands; Ikeda et al. (1998) studied populations in Honshu; and Aotsuka et al. (1995) investigated populations from Kanagawa and Tokyo Prefectures in Honshu.

The current study aims to determine the degree of genetic variability and population structure of *G.dehaani* populations using partial sequences of mitochondrial DNA (mtDNA) from cytochrome oxidase I (*COI*) and cytochrome B (*cytB*) regions from wide geographically distinct localities across the Japanese archipelago from southern Kyush to northern Honshu.

## Material and methods

**Figure 1.**
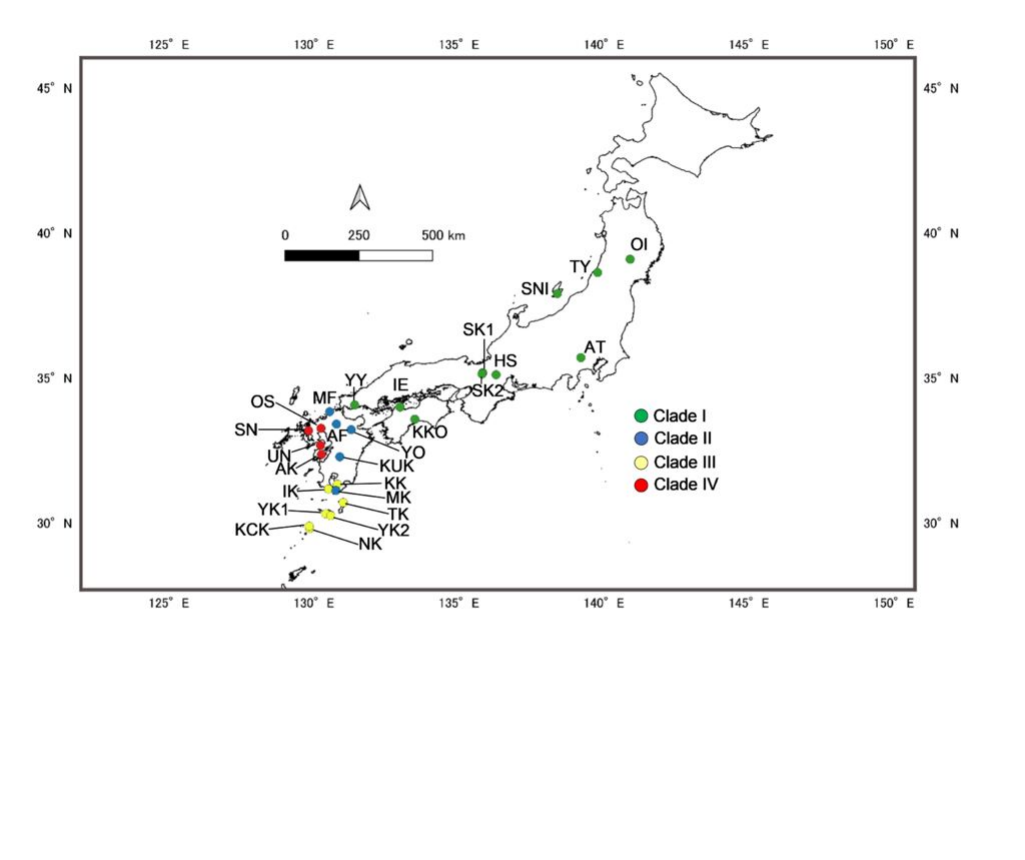
Map showing the locations of 26 *Geothelphusadehaani* populations used in the study and the respective distribution of four mitochondrial clades. Details of the population codes and the corresponding sampling sizes are presented in Table 1.

Two hundred and thirty-one *G.dehaani* specimens used for the analysis of *COI* and *cytB* were obtained from 26 localities covering the Japanese mainland from Honshu to Kyushu and the neighbouring islands of Kyushu (Fig. 1). The areas investigated in the present work include the known distribution range of *G.dehaani* (see Suzuki and Tsuda (1991)). Another species of the same genus *G.marmorata* from Yakushima Island were also sequenced (two specimens) and used as an outgroup.

The *Geothelphusa* specimens used were identified, based on its morphological characters (Chokki 1976; Suzuki and Okano 2000). Body colour types for *G.dehaani* are described as follows: RE – dark brown carapace and reddish legs; DA – dark purplish carapace and legs; and BL – greyish-blue carapace and light-grey legs (see Chokki (1976)). Freshly preserved or live samples were utilised for DNA extraction.

**DNA extraction, amplification and sequencing**

Total DNA was extracted from the leg muscles of each crab using the Quick DNA^TM^ Miniprep Plus Kit (Zymo Research, USA) following the manufacturer’s instructions. Fragments of *COI* and *cytB* genes were amplified using the following primers: *COI* (COIspF: 5ʹ-ATT AGG AGC CCC AGA TAT GGC C-3ʹ and COIorR: 5´-TGG TGA GCT CAT ACT ACA AAT CC-3´) and *cytB* (cytBorF: 5´-ATG ATT TCT CCT ATT CGA AAA TCC C-3´ and cytBotR: 5´-GAT AAA ACA AGG GCT ACA ACT CC-3´). The primers were designed, based on the published mitochondrial genome of *G.dehaani* (GenBank Accession no. AB187570.1: Segawa and Aotsuka (2005)). Amplification of the target genes were carried out in a final reaction volume of 20 µl consisting of 2 µl of each 10 µM primers, 10 µl of EmeraldAmp® GT PCR Master Mix (TaKaRa Bio Inc., Japan), 5 µl milliQ water and 1 µl of DNA template. Amplification was performed in a Bio-RAD T100 ^TM^ thermal cycler (Bio-Rad Laboratories, USA) under the following conditions: 30 s initial denaturation step at 95°C, followed by 30 cycles of 15 s denaturation at 95°C, 15 s annealing at 56°C and 40 s extension at 72°C, then by a final extension step of 1 min at 72°C.

All PCR amplification results were visualised to confirm the presence of the target size product using 1.0% agarose gel stained with ethidium bromide. Prior to sequencing, the PCR products were purified with Agencourt AMPure XP magnetic beads (Beckman Coulter, USA). The sequence primers used for *COI* and *cytB* were similar to the primers in the PCR reaction. Sequencing of the purified PCR products were performed by Fasmac sequencing service (FASMAC, Japan) in an Applied Biosystems 3730xl DNA Analyzer (Thermo Fisher Scientific, USA) and the resulting chromatograms were assessed in Finch TV Version 1.4.0 Geospiza Inc. (Patterson et al. 2006).

The sequences obtained from each gene were verified and aligned with ClustalW implemented in MEGA X software (Kumar et al. 2018). The identical haplotypes from the sequence data of *COI* and *cytB* regions were identified and collapsed using FaBox 1.61 (https://users-birc.au.dk/palle/php/fabox/).

**Data analysis**

The two gene alignments were combined into concatenated data for use in the phylogenetic analysis performed in IQ-TREE 1.6.12 (Nguyen et al. 2015). Phylogenetic tree was constructed by maximum likelihood (ML) method and the reliability of internal branches were assessed by 10,000 ultrafast bootstrap replicates (Hoang et al. 2018). A partition file was prepared and edge-proportional model was applied for the analysis (Chernomor et al. 2016). The best-fit substitution model for each gene partition was selected using ModelFinder under the Bayesian Information Criterion (Schwarz 1978; Kalyaanamoorthy et al. 2017). The suggested best-fit models in each partition were HKY+F+R2 and TPM3+F+G4 for *COI* and *cytB*, respectively.

To investigate the genetic variation within the species of *Geothelphusa* in Japan, the *COI* sequence data of *G.dehaani* and *G.marmorata* from this study and seven other species available in GenBank (accession numbers: AB266313, AB266312, AB625763, AB625762, AB625760, AB625728, LC743300) were used for the ML analysis. The *Geothelphusa* dataset was aligned and analysed following similar steps applied to the *COI* dataset, but the K3Pu+F+I+G4 model was applied as the best-fit model. The endemic potamid of mainland China, Longpotamon (Sinopotamon) xiushuiense (GenBank accession number: NC 029226) was used as an outgroup as it is sister to *Geothelphusa* in the molecular analysis of Wang et al. (2016). The *cytB* gene was not evaluated because there was no available sequence data from other *Geothelphusa* species in the database.

For genetic analyses, the genetic diversity indices i.e. the number of haplotypes (Hn), haplotype diversity (Hd) and nucleotide diversity (π) of each *G.dehaani* population and the four identified clades, based on the combined datasets, were calculated using Arlequin v. 3.5 (Excoffier and Lischer 2010). Pairwise FST values, neutrality tests (Tajima’s D; Fu’s FS), mismatch distribution analysis, based on the sudden expansion model and analysis of molecular variance (AMOVA) were also calculated in Arlequin v. 3.5 (Excoffier and Lischer 2010).

## Results

**Sequence analysis**

The aligned sequence lengths of the *COI* region comprised of 547 base positions (bp) from the 231 *G.dehaani* samples. Eighty-two positions were variable and 79 were parsimony informative. Amongst the total number of sequences, 69 different haplotypes were recorded. The sequence of haplotypes were deposited in DDBJ database under accession numbers LC735417 to LC735485. The nucleotide composition of the *COI* region was AT-rich (63.2%) (A:27.4%, T:35.8%, C:20.5%, G:16.3%). For the *cytB* gene, the 592 bp fragment was amplified, resulting in 46 unique haplotypes. The fragment of *cytB* sequences was also AT-rich (68%) (A:27.4%, T:40.6%, C:18%, G:13.9%). In this region, 75 sites were variable and 74 were parsimony informative. The DDBJ accession number for this gene were LC735488 to LC735533.

When considering the combined fragments of *COI* and *cytB*, a total of 1139 bp were generated and resulted in 93 haplotypes. From the 26 populations, only the crab samples collected from two rivers in Sakyo, Kyoto (SK1 and SK2) shared an identical haplotype (D2) while the remaining populations were characterised with unique haplotypes (Table 1).

**Phylogenetic analysis**

**Figure 2.**
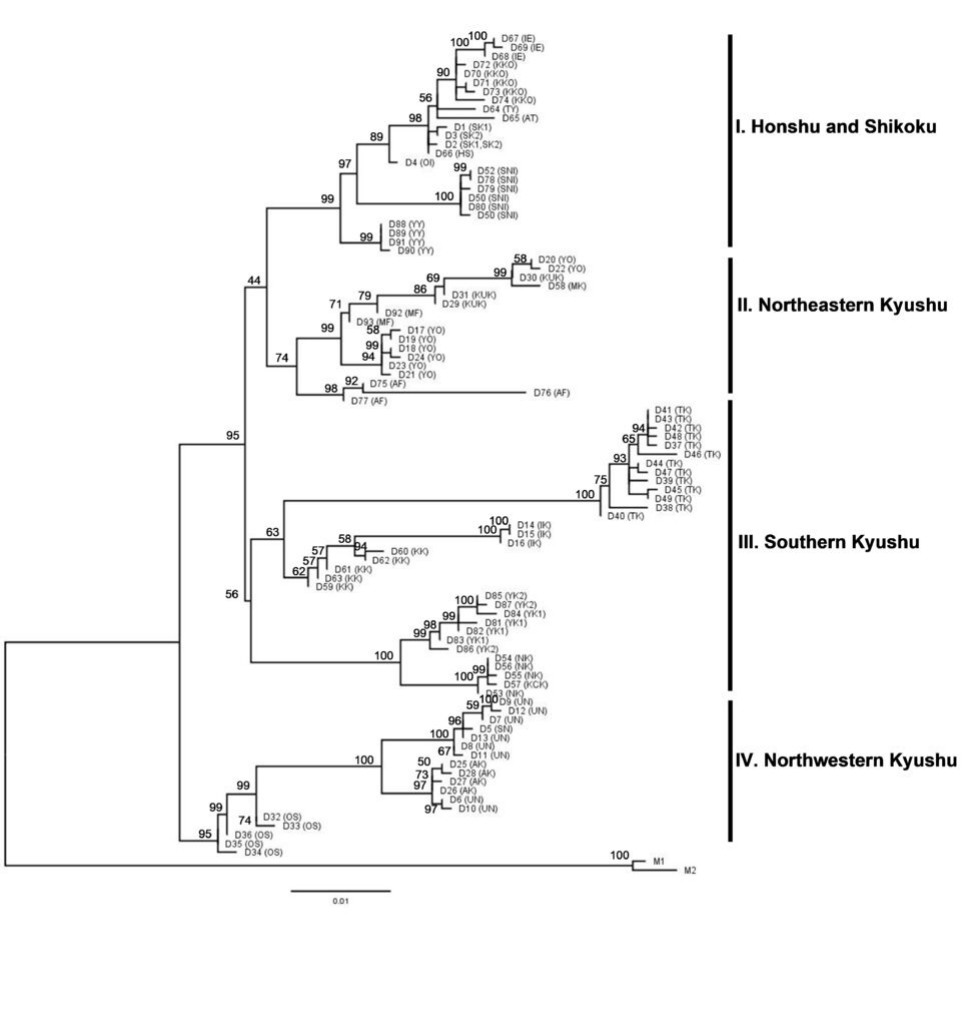
Maximum Likelihood tree of the 93 haplotypes of *Geothelphusadehaani* (D1 to 93), based on the combined *COI* and *cytB* genes. The support of each branch is indicated by percentages on each node. Bar signifies 1% nucleotide sequence difference. Letters in parentheses refer to population code as shown in Table 1. *Geothelphusamarmorata* (M1 and M2) is used as the outgroup.

The phylogenetic tree of the combined *COI* and *cytB* datasets, along with the respective bootstrap values are shown in Fig. 2. The *G dehaani* populations are monophyletic with four geographical groups: Clade I – Honshu and Shikoku; Clade II – north-eastern Kyushu; Clade III – southern Kyushu and Clade IV – north-western Kyushu. Except for Clade III, which had moderate support at 56%, the majority of these clades had high bootstrap values (bs, 74-99%). The northern region of Kyushu was divided into two clades: the eastern clade (II) with Fukuoka, Oita and Kumamoto populations and the western clade (IV) with Nagasaki, Saga and Amakusa populations. The Kagoshima mainland and the Osumi island group (Tanegashima, Yakushima, Nakanoshima and Kuchinoshima) were represented by Clade III. However, the haplotype of Minamiosumi population did not correspond to Clade III, but was more closely related to Clade II.

The monophyly of *G.dehaani* was also demonstrated, based on the *COI* gene with high support value (99%) (Suppl. material 1). The ML tree clearly indicated that this species was distinct from other *Geothelphusa* species found in Japan. *Geothelphusadehaani* was sister clade to *G.marmorata* collected from Yakushima Island and *G.sakamatoana* from Okinawa. The majority of the observed geographical clades were consistent with the result from the combined gene analysis in Fig. 2, but there were some haplotypes that did not correspond to the detected clades. Additionally, the groups from southern Kyushu were not monophyletic.

**Genetic diversity**

Table 1 and Table 2 present the estimated gene diversity indices for each population and the observed clades, respectively. When all populations were considered, UN had the highest haplotype diversity (Hd) (0.972 ± 0.064), followed by TK (0.971 ± 0.039) and OS and KK (0.933 ± 0.122). In terms of nucleotide diversity (π), AF had the highest (0.009 ± 0.005), followed by UN (0.006 ± 0.004) and YO and TK (0.004 ± 0.002). These results indicate that Kyushu populations exhibited higher genetic diversity than Honshu and Shikoku populations. When assessed by clade, the north-western Kyushu clade (Hd: 0.946 ± 0.031; 0.011 ± 0.005) had the highest genetic diversity, followed by the southern Kyushu clade (Hd: 0.942 ± 0.012; π: 0.026 ± 0.013).

Results of the neutrality test and mismatch distributions of the detected clades are shown in Table 3. Tajima’s D was positive in all clades. Two clades had negative values in Fu’s FS test (Clade I: -0.102 and Clade IV: -1.960), of which Clade IV was supported by statistical significance (P < 0.05). In all clades, mismatch distribution analysis revealed that the estimated effective population size after population growth (ϴ_1_) was greater than the estimated effective population size before population growth (ϴ_0_). Clade III had higher ϴ_1_ (65.178) than the other groups with nearly similar values (24.082-26.020). The sum of the square deviations between observed and expected mismatch (SSD) and raggedness index (Rag) were low in all clades and were not statistically significant (P > 0.05). Clade III (49.695) had the highest Tau (τ) estimate compared to other clades (Clade I: 5.869; Clade II: 13.340; Clade IV: 19.609).

The AMOVA results revealed a significant genetic structure across all levels, with 44.01% of the variation occurring from the differences amongst clades, 49.78% of the variation were observed amongst populations within clades and 6.22% of the variation occurred within populations examined (Table 4). The pairwise FST comparisons also revealed significant differentiation (P = 0.000) between each clade (Table 5), with Clade IV notably the most distant from the rest. Clade I and IV had the highest FST value of 0.657, while Clade II and III had the lowest FST value of 0.366. Similarly, regardless of clade classification, high FST values are recorded between each population, indicating a significant degree of differentiation (Suppl. material 2). Low FST values were only observed between SK1 and SK2 (-0.215) and YK1 and YK2 (0.200), with no statistical difference at P = 0.645 and P = 0.080, respectively. These findings can be explained by their close geographic proximity.

## Discussion

The results of phylogenetic analysis of *Geothelphusa* freshwater crabs in Japan, based on the *COI* gene, showed that *G.dehaani* is monophyletic and has the closest relationship with *G.marmorata* from Yakushima Island and *G.sakamotoana* in Okinawa (Suppl. material 1). Together with *G.koshikiensis* from Koshiki Island, they then formed another monophyletic group. This finding indicates that *G.dehaani* has more genetic affinity with other *Geothelphusa* species near the Kyushu mainland. Based on mitochondrial and nuclear DNA markers, *G.sakamatoana* has been reported as a sister species of *G.dehaani* (Lee and Kim 2020). The remaining five species, which are more genetically distant from *G.dehaani*, are mainly concentrated in the Ryukyus.

Using the mtDNA *COI* and *cytB* gene sequences, four distinct geographic groups were identified in the Japanese freshwater crab *G.dehaani* (Clade I – Honshu and Shikoku; Clade II – north-eastern Kyushu; Clade III – southern Kyushu; and Clade IV – North-western Kyushu). Previous analyses have suggested that the ancestor of genus *Geothelphusa* originated from the southern region of continental East Asia and dispersed northwards (Shih and Ng 2011). Based on these findings, this study suggests that the original *G.dehaani* stock first made contact in Kyushu and subsequently migrated northwards to Honshu. During periods of glaciation, the main islands of the Japanese archipelago (Honshu, Shikoku and Kyushu including the Osumi island group) were connected by land bridges to form Paleo-Honshu which allowed for the continuous dispersal of freshwater crab (Yonekura et al. 2001; Iwase et al. 2012). However, geographical features, such as marine waters have been demonstrated to restrict the dispersal of *Geothelphusa* crabs, leading to the confinement of crab populations to certain areas and resulting in regional differentiation (Cumberlidge and Ng 2010; Shih et al. 2011; Shih and Ng 2011). The Seto Inland Sea, formed by marine transgression in the Holocene (11,000 years ago), may have influenced the genetic separation of Clade I (Honshu and Shikoku) from the Kyushu group (Clades II to IV) (Yashima 1994). These marine waters are known to act as barriers to dispersal in freshwater fish and have been implicated in intraspecific divergences in various Japanese freshwater fish (Watanabe et al. 2017). Despite being separated by the Seto Inland Sea, Honshu and Shikoku maintain closer genetic relationships in the present study. This could be due to their relatively recent separation around 7,000 years ago (Ohshima 1990). As a result, the differentiation between the two main islands, based on the *COI* and *cytB* genes, is not yet evident.

In Kyushu, three geographical group were found representing the north-eastern (Clade II), north-western (Clade IV) and southern (Clade III) group. This division apparently resulted from the uneven submergence of areas during the interglacial period and/or major geological events. The Chikushi Plains, currently located between the Sefuri and Sangun Mountains in northern Kyushu, may represent a potential boundary between Clades II and IV. These plains were likely submerged during the interglacial period, which may have contributed to the vicariance of these clades (Sugitani 1983; Tominaga et al. 2006). Subsequently, the sustained isolation in Clade IV over a long period resulted to its significant differentiation from other clades (Avise 2000). Similarly, the high haplotype diversity (0.946 ± 0.031), but low nucleotide diversity (0.011 ± 0.005) in Clade IV suggests that the populations may have experienced rapid population growth from an ancestral population with a small effective population size (Avise 2000). This phenomenon is likely due to various geological processes, such as volcanic activity that caused frequent disturbances in the area, for instance, the series of eruptions in the Unzen Volcano from 150,000 years ago until the present (Hoshizumi et al. 1999; Machida et al. 2001). These multiple volcanic eruptions might have led to a reduction in suitable habitat for *G.dehaani* populations in the area, making them more vulnerable to a decline in population size. This is supported by demographic results, which showed a sudden population expansion in Clade IV after a period of low effective population size (Table 3). The low nucleotide diversities observed may also be a result of the relatively short existence of haplotypes, possibly due to population bottlenecks immediately followed by demographic expansions (Cassone and Boulding 2006). On the other hand, this study did not clearly establish the geographical boundary with respect to the genetic variation between north-eastern Kyushu (Clade II) and southern Kyushu (Clade III). Nonetheless, we propose that Clade III populations may be recent introductions from the common ancestor lineage of *G.dehaani*. This lineage is believed to have dispersed from the Osumi island group (Tanegashima, Yakushima, Nakanoshima and Kuchinoshima) to the Kyushu mainland via land bridges during the late glacial period (about 90,000-10,000 years ago), according to Furukawa and Fujitani (2014). The clustering of the MK population in the Osumi Peninsula with Clade II suggests that this population may have been able to maintain habitable areas during the interglacial period. This could have led to the preservation of genetic characters similar to those found in Clade II. On the contrary, it is possible that the other population in Osumi Peninsula (KK) and in Satsuma Peninsula (IK), were submerged during that period (Ota and Omura 1991). Due to the limited number of populations that were studied in the two main peninsulas of southern Kyushu and the inconsistent findings obtained, more sampling is necessary to clarify this issue.

The AMOVA results provided support for the high levels of structure observed between clades. The total variance analysis indicated that a large genetic variation was present amongst clades (44.01%), populations within clades (49.78%) and a few within populations (6.22%). A previous study on *G.dehaani* revealed that high genetic diversity was observed amongst populations, with a percentage of 63.7%, which is slightly higher than the current results (Ikeda et al. 1998). This pattern of larger genetic differences amongst populations than within them has also been observed in other species of freshwater crabs using an mtDNA marker. For example, in the Chinese potamid *Longpotamonyangtsekiense*, over 50% of the genetic variations are found amongst groups while 32.23% of the variations amongst populations within groups and 13.58% variations within populations (Shi et al. 2021). Similarly, for the Chinese *Longpotamonacutum*, AMOVA showed 72.52% genetic variance between groups and only 9.01% was explained by within-population variance (Fang et al. 2015). High FST values amongst clades and amongst populations also indicate a strong degree of genetic differentiation (Table 5; Suppl. material 2).

The analysis of mitochondrial DNA sequence data showed that *G.dehaani* populations had high haplotype diversity, low nucleotide diversity and a large number of rare haplotypes (Table 1). This pattern is typical of species with limited dispersal ability, which is supported by previous studies on other freshwater species, such as prawns (Liu et al. 2011), fish (Dodson et al. 1995; Garg and Mishra 2018) and crabs (Shih et al. 2004; Shih et al. 2006; Fang et al. 2015; Shi et al. 2021). The presence of unique haplotypes suggests that *G.dehaani* populations are geographically isolated, with little gene flow occurring between them (Avise 1994). The shared haplotype in SK populations was likely due to the close proximity of the collection site. *Geothelphusadehaani* is an amphibious crab and can survive desiccation for days, thus can disperse to short distances across land especially during the rainy season (Okano et al. 2000a; Batang and Suzuki 2003; Okano et al. 2003).

The Japanese freshwater crab *G.dehaani* has three different colour types: RE, which has a dark brown carapace and reddish legs; DA, which has a dark purplish carapace and legs; and BL, which has a greyish-blue carapace and light-grey legs (Chokki 1976). Apparently, the causative genetic or biological mechanisms behind these colour variations are still not well understood. Aotsuka et al. (1995) reported that DA and BL colour populations in Kanagawa Prefecture of Honshu existed with substantial genetic variations, based on allele frequencies. A similar study was also evaluated by Ikeda et al. (1998) in Honshu, but with a wider coverage area where they found that only the BL type was distantly related to other types, while the genetic differentiation of RE and DA was not supported as identified by electrophoretic analysis. Okano et al. (2000b) noted that there was no genetic differentiation observed amongst the different colour types in Kagoshima mainland. In this study, we were also unable to establish any connections between body colouration and genetic variations. All three colour types were found in Clade I, RE and BL types were found in Clade II, probably only the DA type in Clade III and only the RE type in Clade IV (Table 1).

In conclusion, this study found that *G.dehaani* can be divided into four distinct geographical clades, based on *COI* and *cytB* datasets. High genetic variations were observed amongst the populations examined as a result of low gene flow. The geologically dynamic history of the Japanese archipelago appears to have sporadically and repeatedly facilitated discontinuity in freshwater habitats over evolutionary time, thereby inducing strong divergence amongst populations of *G.dehaani*. The findings highlight the need for further examination of morphological and behavioural characteristics in the populations studied in Kyushu. Future research should involve more extensive sampling and the use of advanced molecular tools like next-generation sequencing.

## Supplementary Material

0DBC734A-5624-55B9-BCC5-6937E978893D 10.3897/BDJ.11.e97438.suppl1 Supplementary material 1 Maximum Likelihood tree Data type pdf Brief description Maximum Likelihood tree showing the relationships between haplotypes of 26 *Geothelphusadehaani* populations and other *Geothelphusa* species in Japan, based on *COI* gene. File: oo_834587.pdf https://binary.pensoft.net/file/834587 Joana Joy de la Cruz-Huervana

02BC5A11-454B-5BCB-87C8-1D31D405A0A3 10.3897/BDJ.11.e97438.suppl2 Supplementary material 2 Matrix of pairwise differences of FST Data type xlsx Brief description Matrix of pairwise differences of FST amongst populations of *Geothelphusadehaani* using the combined *COI* and *cytB* sequences. File: oo_799932.xlsx https://binary.pensoft.net/file/799932 Joana Joy de la Cruz-Huervana

## Figures and Tables

**Table 1.** Genetic diversity indices of *Geothelphusadehaani* collected from 26 populations using the combined *COI* and *cytB* genes.

**Pop code**	**Location**	**Region**	**Body colour**	**N**	**Hn**	**Haplotype number**	**Hd (sd)**	**π (sd)**
Clade I								
OI	Oshu, Iwate	Honshu	DA	13	1	D4	0	0
HS	Higashiomi, Shiga	Honshu	RE	10	1	D66	0	0
SK1	Sakyo, Kyoto	Honshu	RE	6	2	D1,2	0.533±0.172	0.001±0.001
SK2	Sakyo, Kyoto	Honshu	RE	3	2	D2,3	0.667±0.314	0.001±0.001
TY	Tsuruoka, Yamagata	Honshu	DA	3	1	D64	0	0
AT	Akiruno, Tokyo	Honshu	DA	7	1	D65	0	0
SNI	Sado, Niigata	Honshu	BL	18	6	D50,51,52, 78,79,80	0.817±0.054	0.001±0.000
YY	Yamaguchi	Honshu	-	6	4	D88,89,90,91	0.867±0.129	0.001±0.000
KK0	Kochi, Kochi	Shikoku	RE	12	5	D70,71,72,73, 74	0.727±0.113	0.001±0.001
IE	Imabari, Ehime	Shikoku	RE	16	3	D67,68,69	0.425±0.133	0.000±0.000
Clade II								
AF	Asakura, Fukuoka	North-eastern Kyushu	RE	8	3	D75,76,77	0.679±0.122	0.009±0.005
MF	Munakata, Fukuoka	North-eastern Kyushu	RE	8	2	D92,93	0.429±0.169	0.001±0.001
KUK	Kuma, Kumamoto	North-eastern Kyushu	RE	9	3	D29,30,31	0.556±0.165	0.005±0.003
YO	Yufu, Oita	North-eastern Kyushu	RE	12	8	D17,18,19,20, 21,22,23,24	0.909±0.065	0.004±0.002
MK	Minamiosumi, Kagoshima	Southern Kyushu	BL	5	1	D58	0	0
Clade III								
KK	Kanoya, Kagoshima	Southern Kyushu	-	6	5	D59,60,61, 62,63	0.933±0.122	0.003±0.002
IK	Ibusuki, Kagoshima	Southern Kyushu	DA	10	3	D14,15,16	0.378±0.181	0.0003±0.0004
TK	Tanegashima Island, Kagoshima	Southern Kyushu	DA	15	13	D37,38,39, 40,41,42,43, 44,45,46,47, 48,49	0.971±0.039	0.004±0.002
YK1	Yakushima Island, Kagoshima	Southern Kyushu	-	6	4	D81,82,83,84	0.800±0.172	0.002±0.002
YK2	Yakushima Island, Kagoshima	Southern Kyushu	-	6	3	D85,86,87	0.600±0.215	0.002±0.002
NK	Nakanoshima Island, Kagoshima	Southern Kyushu	DA	16	4	D53,54,55,56	0.725±0.074	0.001±0.001
KCK	Kuchinoshima Island, Kagoshima	Southern Kyushu	DA	9	1	D57	0	0
Clade IV								
OS	Ogi, Saga	North-western Kyushu	RE	6	5	D32,33,34, 35,36	0.933±0.122	0.003±0.002
AK	Amakusa, Kumamoto	North-western Kyushu	RE	6	4	D25,26,27,28	0.867±0.129	0.001±0.001
UN	Unzen, Nagasaki	North-western Kyushu	RE	9	8	D6,7,8,9,10, 11,12,13	0.972±0.064	0.006±0.004
SN	Sasebo, Nagasaki	North-western Kyushu	RE	6	1	D5	0	0

N: number of specimens; Hn: number of observed haplotypes; Hd: haplotype diversity; π: nucleotide diversity; sd: standard deviation; RE – dark brown carapace and reddish legs; DA – dark purplish carapace and legs; BL – greyish-blue carapace and light-grey legs (see Chokki (1976)); Hyphen (-) indicates no data.

**Table 2.** Genetic diversity indices of the clades detected in *Geothelphusadehaani* using the combined *COI* and *cytB* genes.

Clade	N	Hn	Hd (sd)	π (sd)
I	94	25	0.936 ± 0.010	0.009 ± 0.005
II	42	17	0.934 ± 0.017	0.012 ± 0.006
III	68	33	0.942 ± 0.012	0.026 ± 0.013
IV	27	18	0.946 ± 0.031	0.011 ± 0.005

N: number of specimens; Hn: number of observed haplotypes; Hd: haplotype diversity; π: nucleotide diversity; sd: standard deviation.

**Table 3.** Neutrality data and mismatch distribution analysis of the three clades detected in *Geothelphusadehaani*, using the combined *COI* and *cytB* genes.

Clade	Tajima’s D	Fu’s FS	τ	ϴ_0_	ϴ_1_	SSD	Rag
I	0.288	-0.102	5.869	9.865	24.082	0.012	0.016
II	1.161	1.875	13.340	2.809	26.020	0.016	0.021
III	2.044	1.361	49.695	0.000	65.178	0.014	0.014
IV	1.556	-1.960*	19.609	0.000	24.201	0.019	0.013
Mean	1.262	0.293	22.128	3.168	34.870	0.015	0.016

τ: Tau; ϴ_0_: ϴ before population growth; ϴ_1_: ϴ after population growth; SSD: sum of the square deviations between the observed and expected mismatch; Rag: raggedness index; Asterisk (*) indicates P < 0.05.

**Table 4.** Analysis of molecular variance (AMOVA) for the combined *COI* and *cytB* sequences of *Geothelphusadehaani*.

Source of variation	d.f.	Sum of squares	Variance components	Percentage of variation	Fixation indices	P-value
Among clades	3	1503.511	7.718Va	44.01	Fsc = 0.889	0.000
Among populations within clades	22	1697.003	8.730Vb	49.78	Fst = 0.938	0.000
Within populations	205	223.551	1.090Vc	6.22	Fct = 0.440	0.000
Total	230	3424.065	17.539			

**Table 5.** Matrix of pairwise differences of FST of the four clades detected in *Geothelphusadehaani*, using the combined COI and cytB genes.

Clade	I	II	III	IV
I	0.000			
II	0.626*	0.000		
III	0.483*	0.366*	0.000	
IV	0.657*	0.627*	0.462*	0.000

Asterisk (*) indicates P=0.000.
