# Supplementary material for: Preliminary assessment of genetic variation in the Japanese endemic freshwater crab, Geothelphusadehaani, based on mitochondrial DNA sequences
Source: Biodivers Data J. 2023 Jun 27;11:e97438. doi: 10.3897/BDJ.11.e97438 (PMC10848686; doi:10.3897/BDJ.11.e97438)
Supplement: Supplementary material 1 — Maximum Likelihood tree [file bdj-11-e97438-s001.pdf]

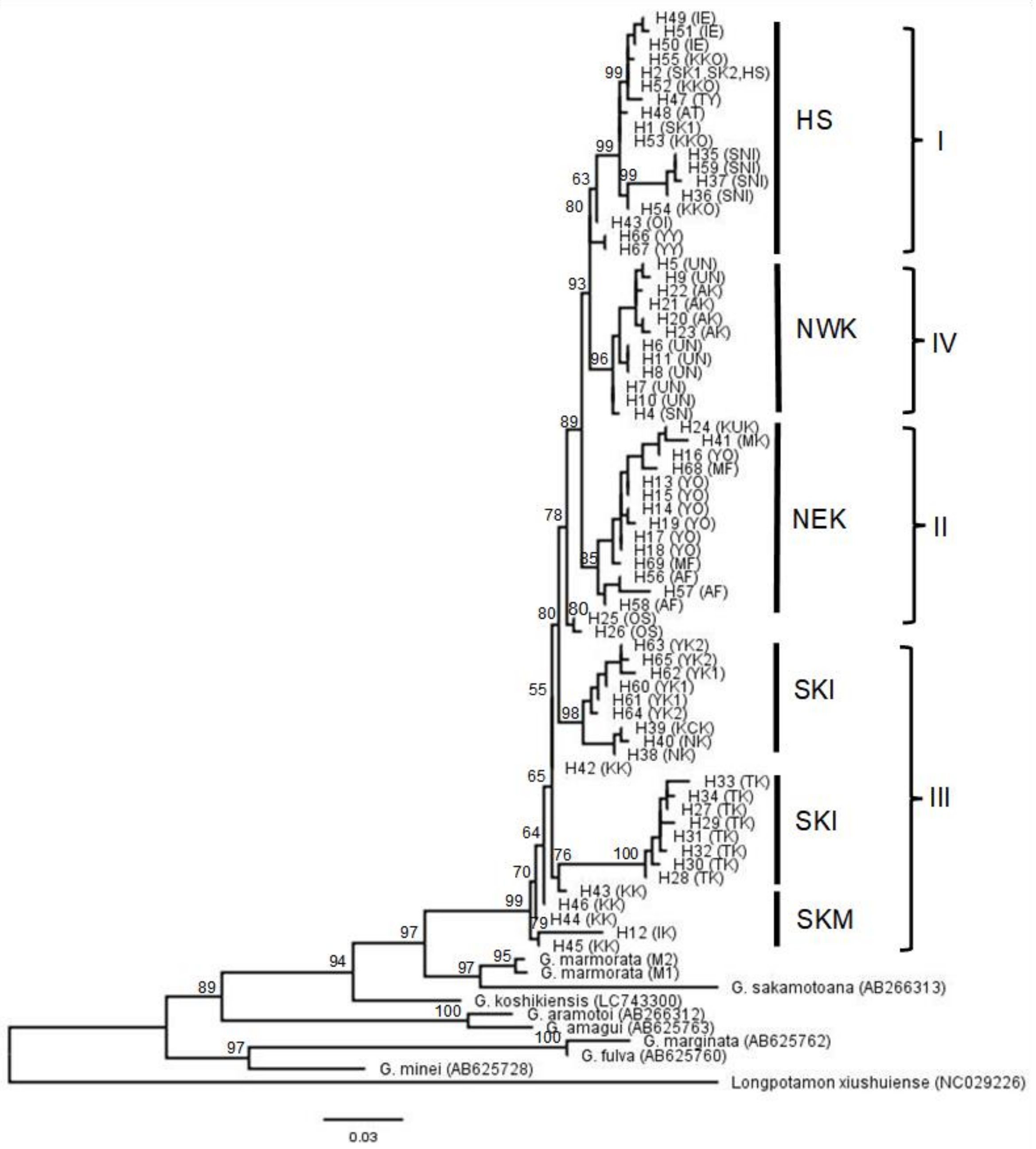


Suppl. material 1. Maximum likelihood tree showing the relationships between haplotypes of 26 *Geothelphusa dehaani* populations and other *Geothelphusa* species in Japan based on *COI* gene. The support of each branch is indicated by percentages on each node and bar signifies nucleotide sequence difference. Letters in parentheses refer to population code as shown in Table 1. H, haplotypes of *G. dehaani*; M, haplotypes of *G. marmorata*; *Longpotamon* (*Sinopotamon*) *xiushuiense* (outgroup); HS, Honshu and Shikoku; NWK, Northwestern Kyushu; NEK, Northeastern Kyushu; SKM, Southern Kyushu Mainland; SKI, Southern Kyushu Island.
